# Selective Dorsal Rhizotomy For Treatment of Spasticity After Hemispherectomy In Children: A Case Report

**DOI:** 10.7759/cureus.36945

**Published:** 2023-03-31

**Authors:** TS Park, Susan Joh, Matthew D Smyth, Nicole L Meyer, Deanna M Walter

**Affiliations:** 1 Neurological Surgery, Washington University School of Medicine, St. Louis Children's Hospital, St. Louis, USA; 2 Pediatric Neurosurgery, Washington University School of Medicine, St. Louis Children's Hospital, St. Louis, USA

**Keywords:** selective dorsal rhizotomy, hemispherectomy, hemispherotomy, muscle spasticity, hemiparesis, epilepsy, muscle tone

## Abstract

Performing a hemispherotomy or hemispherectomy is known to treat medically intractable epilepsy successfully, yet contralateral hemiparesis and increased muscle tone follow the epilepsy surgery. Spasticity and coexisting dystonia presumably cause the increased muscle tone in the lower extremity on the opposite side of epilepsy surgery. However, the extent of the role of spasticity and dystonia in high muscle tone is unknown. A selective dorsal rhizotomy is performed to reduce spasticity. If a selective dorsal rhizotomy is performed in the affected patient and muscle tone is reduced, the high muscle tone is not due to dystonia. Two children, who previously underwent a hemispherectomy or hemispherotomy, had a selective dorsal rhizotomy (SDR) performed in our clinic. Both children underwent orthopedic surgery to treat heel cord contractures. To study the extent of the role of spasticity and dystonia in high muscle tone, the mobility of the two children was examined pre- and post-SDR. The children had follow-ups 12 months and 56 months after SDR to study long-term effects. Before SDR, both children showed signs of spasticity. The SDR procedure removed spasticity, and muscle tone in the lower extremity became normal. Importantly, dystonia did not surface after SDR. Patients started independent walking less than two weeks after SDR. Sitting, standing, walking, and balance improved. They could walk longer distances while experiencing less fatigue. Running, jumping, and other more vigorous physical activities became possible. Notably, one child showed voluntary foot dorsiflexion that was absent before SDR. The other child showed improvement in voluntary foot dorsiflexion that was present before SDR. Both children maintained the progress at the 12 and 56-month follow-up visits. The SDR procedure normalized muscle tone and improved ambulation by removing spasticity. The high muscle tone following the epilepsy surgery was not due to dystonia.

## Introduction

The hemispherectomy or hemispherotomy is widely utilized to treat intractable epilepsy [1-3]. However, high muscle tone on the opposite side follows the epilepsy surgery and hinders ambulation after the procedure. Spasticity and dystonia are possible causes, but the role of dystonia in increased muscle tone is unclear. SDR can eliminate spasticity [4,5]. The unique effect of the surgery allows for identifying the cause of altered muscle tone after epilepsy surgery. In individual patients, normalization of muscle tone after SDR would point to spasticity as the primary cause of the increased muscle tone. We report details of two children whose increased lower limb muscle tone after epilepsy surgery resulted solely from spasticity. Additionally, SDR improved independent ambulation in both children, and dystonia was absent.

## Case presentation

Patient one

History

This female was born full-term with left ventriculomegaly. A congenital brain malformation was suspected based on an ultrasound. At five years of age, she had status epilepticus complicated by new right hemiparesis. By ten years of age, her seizures became medically refractory. Accordingly, she underwent a comprehensive evaluation to determine her candidacy for epilepsy surgery.

Neuropsychologic Test

She showed left lateralizing dysfunction with a split between verbal reasoning and visual perceptual reasoning. She had multiple deficits in language functioning. 

*Electroencephalogram* (*EEG) Video Monitoring*

Noninvasive monitoring showed marked asymmetry of the background activities with lower amplitude left-hemispheric activities most prominent in the left frontal region. Seizures had unequivocal electrographic onset in the left occipital region. 

MRI and Positron Emission Tomography (PET)

The preoperative MRI revealed atrophy of the left cerebral hemisphere, dilated left lateral ventricle, and left mesial temporal lobe sclerosis. The functional MRI indicated an expressive language center in the right frontal and temporal lobes and a comprehensive language center in the left temporal lobe. In addition, FDG-PET (fluorodeoxyglucose positron emission tomography) showed decreased FDG uptake in the entire left cerebral hemisphere and the right mesial temporal lobe. 

Epilepsy Surgery

Based on the evaluation results, our epilepsy team recommended a left-sided hemispherotomy. She underwent surgery at ten years of age. Pathology of the resected brain was cortical dysplasia, neuronal loss, gliosis, and mesial temporal gliosis. Postoperatively, she became seizure-free while taking only Keppra. Speech function remained intact.

Selective Dorsal Rhizotomy 

She resumed independent walking after recuperating from the hemispherotomy. But spasticity and hemiplegic gait were worse than before the hemispherotomy. Thus, the parents sought an SDR evaluation eighteen months after the hemispherotomy. Evaluation for SDR revealed increased muscle tone, hyperreflexia, ankle clonus, stiff movement, toe walking due to Achilles tendon contracture, and in-toeing in the right lower extremity. Of note is that she could not voluntarily dorsiflex the right foot. Also, she had a leg length discrepancy, with a shorter right lower extremity. By contrast, the left lower extremity had no abnormality.

Sixteen months after the hemispherotomy, she underwent right-sided SDR to remove spasticity. In addition, four months after SDR, she received orthopedic surgeries, including right Achilles tendon lengthening to treat tendon contracture and left distal femoral epiphysiodesis to treat leg length discrepancy. 

*SDR + Orthopedic Surgery Outcomes* SDR eliminated spasticity without an adverse effect. There was no sensory loss or incontinence. She resumed independent walking a week after surgery. Muscle hypertonicity, hyperreflexia, and ankle clonus in the right lower extremity disappeared. Sitting, standing, walking, and balance improved (Video 1).

**Video 1:** Patient 1, walking before SDR Permission was given by the patient to display patient identifying information in the video.

After SDR, she could walk longer distances with less fatigue. She could do vigorous physical activities and exercise (Video 2).

**Video 2:** Patient 1, walking after SDR Permission was given by the patient to display patient identifying information in the video.

She could voluntarily dorsiflex her right foot after SDR, which was not present before SDR (Video 3).

**Video 3:** Patient 1, new voluntary foot movement after SDR Permission was given by the patient to display patient identifying information in the video.

There was no sign of dystonia. Four years and eight months after SDR, she maintained normal muscle tone and improved motor function in the right lower extremity.

Patient two

This boy was born full term. He had an uneventful course until he began having seizures at nine months. The seizure episode was rapidly progressive, ultimately occurring every thirty seconds. He lost his achieved skills at that stage, i.e., sitting, crawling, and nearly walking. He was diagnosed at that time with Sturge-Weber syndrome. He underwent a right-sided hemispherectomy at twelve months of age in the United Kingdom. Since the surgery, he has been seizure-free. Two weeks after the epilepsy surgery, he started sitting, and about four months after surgery, he started walking. He has been ambulating independently since that time with left-sided spastic hemiparesis. 

At four years of age and three years after the hemispherotomy, he underwent evaluation for SDR to treat spasticity. The extremities on the right were normal. But the left-side extremities were affected with spastic weakness, increased muscle tone during voluntary movement, hyperreflexia, and ankle clonus. He could walk independently, but he showed a circumduction gait and impaired balance in walking. In addition, he lacked a left-arm swing (Video 4).

**Video 4:** Patient 2, walking before SDR Permission was given by the patient's parent to display patient identifying information in the video.

Finally, he developed severe heel cord contracture restricting foot dorsiflexion. However, he had some voluntary dorsiflexion of the left foot while lacking toe movements. 

Three years after the right-sided hemispherectomy, he underwent unilateral SDR on the left side. Two weeks after SDR, he received an Achilles tendon lengthening to treat the contracture. He resumed independent walking a week after SDR. He continued to show improvements in independent walking during a follow-up in twelve months of SDR without adverse effects from SDR. He showed no sign of dystonia (Videos 5, 6).

**Video 5:** Patient 2, walking after SDR Permission was given by the patient's parent to display patient identifying information in the video.

**Video 6:** Patient 2, running after SDR Permission was given by the patient's parent to display patient identifying information in the video.

## Discussion

There was no prior report of SDR on patients who underwent ablative epilepsy surgery. In the present article, we provide the clinical details of two patients. The novel experience with our patients is as follows. 1) The hypertonicity of the lower limb in the children was entirely due to spasticity. 2) SDR could eliminate spasticity and normalize muscle tone in the lower extremities. 3) SDR significantly improved the quality of hemiplegic gait. 4) Neither of the children showed dystonia after SDR, ruling out the role of dystonia in post-hemispherotomy hypertonicity. 5) Voluntary foot dorsiflexion on the affected lower extremity emerged or improved after SDR. 

The significance of the absence of dystonia in our patients is twofold. First, the outcome supports the notion that the cerebral cortex is the site of genesis of dystonia. Previous authors stated that dystonia results from insufficient suppression of undesired movements either during rest or during the execution of a particular task. [6,7] Second, the outcome of our patients expands the treatment options for children undergoing hemispherectomy. SDR can remove spasticity, improve walking, allow for exercise, and improve other quality-of-life functions following the epilepsy surgery. The positive outcome of the two children is consistent with the children who had SDR for spastic hemiplegia due to other causes [8].

An unexpected positive outcome in the two children was the emergence or improvement of preexisting voluntary foot dorsiflexion after SDR. The improved foot movement following SDR indicates that spasticity suppressed motor functions. In general, voluntary foot movement results in better ambulation. 

Regarding patient selection for SDR, we performed SDR on seven children of this patient cohort-five with hemispherotomy and two with hemispherectomy. All children were free of seizures for at least twelve months either without or with a single seizure medication. Of significance is that three of the seven children had spasticity in both lower extremities, and bilateral SDR was necessary.

## Conclusions

The two children in the present report had increased muscle tone and hemiparesis contralateral to the hemispherectomy and hemispherotomy epilepsy surgery. Signs of spasticity, namely hyperreflexia and ankle clonus, were present. The symptoms of dystonia were not evident. They underwent unilateral SDR at the L1-S2 level to treat spasticity. We made several novel observations in our patients: The SDR removed spasticity, and muscle tone in the lower extremity became normal. Children could walk longer distances while experiencing less fatigue. Running, jumping, and other more vigorous physical activities became possible. Of particular interest is that one child showed new voluntary foot dorsiflexion absent before SDR. The other child showed improvement in voluntary foot dorsiflexion present before SDR. Significantly, dystonia did not manifest after SDR in our patients.

The present case report suggests that SDR may improve ambulatory functions and the quality of life of children undergoing ablative epilepsy surgery. The utility of SDR for this particular cohort would be an excellent topic for a future multicenter prospective study. 
